# Risk factors and prognosis for neonatal sepsis in southeastern Mexico: analysis of a four-year historic cohort follow-up

**DOI:** 10.1186/1471-2393-12-48

**Published:** 2012-06-12

**Authors:** Yelda A Leal, José Álvarez-Nemegyei, Juan R Velázquez, Ulises Rosado-Quiab, Nidia Diego-Rodríguez, Etna Paz-Baeza, Jorge Dávila-Velázquez

**Affiliations:** 1 Unidad de Investigación Médica Yucatán (UIMY), Unidad Médica de Alta Especialidad de Mérida, Instituto Mexicano del Seguro Social (IMSS), Calle 34 #439 x 41, Col. Industrial (Ex-terrenos del Fénix, Hospital T1), 97150, Mérida, Yucatán, México; 2 Facultad de Medicina Universidad Anáhuac Mayab Mérida, Yucatán, México; 3 Departamento de Inmunogenética y Alergia, Instituto Nacional de Enfermedades Respiratorias (INER), Ciudad de México, México; 4 Coordinación de Vigilancia Epidemiológica, Unidad de Salud Pública, Dirección de Prestaciones Médicas; IMSS, Ciudad de México, México; 5 Servicio de Neonatología de la Unidad Médica de Alta Especialidad de Mérida, IMSS, Mérida, Yucatán, México; 6 Cunero Patológico, Hospital General Regional #1, IMSS, Mérida, Yucatán, México

**Keywords:** Neonatal sepsis, Early-onset neonatal sepsis, Late-onset neonatal sepsis, Risk factors, Prognostic factors, Mortality

## Abstract

****Background**:**

Neonatal sepsis is a worldwide public health issue in which, depending on the studied population, marked variations concerning its risk and prognostic factors have been reported. The aim of this study was to assess risk and prognostic factors for neonatal sepsis prevailing at a medical unit in southeastern Mexico. Thus, we used a historic cohort design to assess the association between a series of neonates and their mothers, in addition to hospital evolution features and the risk and prognosis of neonatal sepsis (defined by Pediatric Sepsis Consensus [PSC] criteria) in 11,790 newborns consecutively admitted to a Neonatology Service in Mérida, Mexico, between 2004 and 2007.

****Results**:**

Sepsis was found in 514 of 11,790 (4.3 %) newborns; 387 of these cases were categorized as early-onset (<72 h) (75.3 %) and 127, as late-onset (>72 h) (24.7 %). After logistic regression, risk factors for sepsis included the following: low birth weight; prematurity; abnormal amniotic fluid; premature membrane rupture (PMR) at >24 h; respiratory complications, and the requirement of assisted ventilation, O_2_ Inspiration fraction (IF) >60 %, or a surgical procedure. Some of these factors were differentially associated with early- or late-onset neonatal sepsis. The overall mortality rate of sepsis was 9.5 %. A marked difference in the mortality rate was found between early- and late-onset sepsis (p >0.0001). After Cox analysis, factors associated with mortality in newborns with sepsis comprised the following: prematurity; low birth weight; low Apgar score; perinatal asphyxia, and the requirement of any invasive medical or surgical procedure.

****Conclusions**:**

The incidence of neonatal sepsis in southeastern Mexico was 4.3 %. A different risk and prognostic profile between early- and late-onset neonatal sepsis was found.

## **Background**

Worldwide, the neonatal mortality rate has been set at 30/1,000, resulting in 4 million deaths each year; 95 % of these deaths occurs in developing countries, where the risk of neonatal death is 6 times higher than that of developed countries [1-3]. The leading causes of neonatal mortality are infections, followed by asphyxia and congenital abnormalities [4-6]. Thus, neonatal sepsis is a relevant public health issue because it consistently emerges as one of the main causes of neonatal morbidity and mortality.

In Mexico, the National Institute for Statistics and Geography (INEGI) reported that neonatal diseases were the first cause (49.8 %) of deaths in children <1 year of age, neonatal sepsis was again found as one of its main specific causes [7,8], and the incidence of neonatal sepsis in 2003 was reported as between 4 and 14 15.4/1,000 live newborns [9]. In Yucatán, a state in southeastern Mexico, a study performed between 2000 and 2004 at the Neonatal Intensive Care Unit of the Mexican Institute of Social Security (IMSS) in Mérida, the neonatal mortality rate was 10.3/1,000 [10].

Despite recent advances in neonatal care, the impact of neonatal sepsis remains marked in developing countries [1,11,12]. Thus, identifying the risk and prognosis factors prevailing in the different geographical contexts has become a crucial issue for optimizing neonatal care. Because there is scarce information on these topics in the geographical context in which the study was carried out, we performed a retrospective study in newborns between 2004 and 2007 that was aimed at assessing the association between neonates and maternal clinical and demographical factors, in addition to clinical course features and the risk for neonatal sepsis.

We categorized sepsis as early-onset when the diagnosis was performed prior to 72 h in the life of the neonate and late-onset if the diagnosis was carried out after this deadline. Usually, early-onset neonatal sepsis is associated with perinatal background and late-onset is mainly related with the medical and surgical invasive procedures required by neonates who already have the disease [4,13-15]. Our results supported this statement, along with an interesting higher death risk in late-onset neonatal sepsis.

## **Results**

### **Studied population**

A total of 11,790 newborns (males, 6,013; females: 5,775; two, unclassified gender) were admitted to the participating Neonatology wards during the study period. The entire cohort totaled 50,900 follow-up days and 4.2 ± 14.6 follow-up days per patient (range, 1–142 follow-up days).

Sepsis was found in 514 (4.3 %) neonates, and appeared as early-onset in 387 and as late-onset in 127 neonates. Sepsis, defined as the microbial isolation of any biological sample, was found in 87 neonates (16.9 %); 9 were from early-onset and 78 from late-onset sepsis. The microbiological profile was as follows: Gram-positive bacteria [mainly Coagulase-Negative *Staphylococcus* Group (*CoNS*)] were identified in 59 (67.8 %) patients; Gram-negative bacteria (mainly *Klebsiella spp.*) were isolated in 15 (17.2 %), and *Candida spp.* was found in 13 (14.9 %) patients (Table 1).

**Table 1:** Microbiological profile found in blood culture from neonates with early- and late-onset sepsis

Isolated microorganism	Early- onset	Late- onset
	N = 387	N = 127
*Escherichia coli*	1	2
*Klebsiella pneumoniae*	1	4
*Enterobacter cloacae*	0	3
*Pseudomonas spp.*	0	3
*Haemophilus influenzae*	0	1
*Streptococcus group B*	2	2
*Staphylococcus aureus*	0	13
*Staphylococcus CoNS*	5	37
*Candida spp.*	0	13
**Total**	**9**	**78**

Neonates with sepsis accounted for 12,884 follow-up days (mean, 25.0 ± 29.3 follow-up days per patient; range, 1–277 follow-up days), which was significantly longer when compared with neonates without sepsis (3.3 ± 12.6 patient/day) p <0.0001.

The final outcome in the entire group of neonates with sepsis was as follows: 444 (86.4 %) were rated as healthy neonates, 21 (4.1 %) were discharged from the hospital with some type of residual damage, and 49 (9.5 %) died. Among neonates with early-onset sepsis, 353 were discharged from the hospital as healthy neonates, 12 (3.1 %) had some type of sequelae, and a fatal outcome was registered in 22 (5.7 %) neonates. On the other hand, prognosis of late-onset neonatal sepsis was poorer because death was registered in 27 (21.3 %) neonates (p <0.0001), 91 (71.7 %) were discharged from the hospital as healthy neonates, and nine (7.1 %) neonates left the hospital with some type of residual damage. Furthermore, neonates with late-onset sepsis had longer hospital stays (48.1 ± 39.0 days) as compared with those of neonates with early-onset sepsis (17.4 ± 19.9 days) p <0.0001.

### **Risk assessment for neonatal sepsis**

Comparison of the clinical and demographic features of the newborns, their mothers, and clinical-course characteristics by univariate analysis between the entire group of neonates with and without sepsis is depicted in Table 2. Of all of these, only neonate gender, post maturity, low maternal educative level, and preeclampsia appearing after 35 gestational weeks were not entered into the multivariable analysis. After logistic regression analysis, only low birth weight (≤2,500 g) RR = 1.0, 95 % CI = 1.0-1.0, p ≤ 0.0001; prematurity (≤37 wks) RR = 2.4, 95 % CI = 1.7-3.4, p ≤ 0.0001; abnormal amniotic fluid RR = 1.5, 95 % CI = 1.1-1.9, p ≤ 0.005; premature membrane rupture (PMR) >24 h RR = 3.5, 95 % CI = 1.8-6.6, p ≤ 0.0001; the emergence of any respiratory complication after birth RR = 35.0, 95 % CI = 22.1-57.1, p ≤ 0.001; and the requirements of assisted ventilation RR = 1.7, 95 % CI = 1.1-2.5, p ≤ 0.004; O_2_IF >60 % RR = 3.2, 95 % CI = 2.1-4.9; p ≤ 0.001; or any type of surgical procedure RR = 9.6, 95 % CI = 3.3-27.7, p ≤ 0.0001; were identified as independent risk factors for sepsis.

**Table 2:** Comparison of all collected data between neonates with and without sepsis (univariate analysis)

**Variable**	**With sepsis**	**Without sepsis**	**P**
	**N = 514 (%)**	**N = 11276 (%)**	
***Neonate-related***			
Gender (feminine/masculine)	233/281	5542/5732	0.22
Gestational age (wk)*	34 ± 4.3	39 ± 2.3	<0.0001
Birth weight (g)*	2069 ± 837	3053 ± 576	<0.0001
Height (cm)*	43.4 ± 5.9	49.3 ± 3.3	<0.0001
Premature (≤37 wks)	350 (68.1)	1581 (14.0)	<0.0001
Postmature (≥42 wks)	5 (1.0)	131 (1.2)	0.45
Product of multiple pregnancy	41 (8.0)	204 (1.8)	<0.0001
Abnormal umbilical cord	131 (25.5)	1620 (14.4)	<0.0001
With perinatal asphyxia	17 (3.3)	81 (0.7)	<0.0001
With intrauterine hypoxia	10 (1.9)	71 (0.6)	0.003
Apgar score ≤5	274 (53.3)	1,765 (15.7)	<0.0001
With fetal distress	86 (16.7)	896 (7.9)	<0.0001
***Maternal-related***			
Age (yrs)*	26 ± 5.5	25 ± 5.4	0.10
Low educative level	284 (55.3)	6341 (56.2)	0.341
Gestations*	2 ± 1.2	2 ± 1.1	0.01
Deliveries*	0.5 ± 0.9	0.6 ± 0.9	0.001
Non-adequate pregnancy control	258 (50.2)	4112 (36.5)	<0.0001
Experienced threat of abortion during this pregnancy	139 (27.0)	1905 (16.9)	<0.0001
Abnormal placenta	86 (16.7)	436 (3.9)	<0.0001
Dystocic delivery	151 (24.9)	5900 (52.3)	<0.0001
Cesarean section	359 (69.8)	5232 (46.4)	<0.0001
Abnormal amniotic fluid	159 (30.9)	1687 (15.0)	<0.0001
Premature membrane rapture > 12 h	168 (32.7)	469 (4.2)	<0.0001
Premature membrane rapture > 24 h	137 (26.7)	222 (2.0)	<0.0001
Premature membrane rapture > 48 h	85 (26.7)	105 (0.9)	<0.0001
Amnionitis	4 (0.8)	32 (0.3)	0.074
Preeclampsia ≤35 gestational wks	28 (5.4)	340 (3.0)	<0.0001
Preeclampsia >35 gestational wks	26 (5.1)	569 (5.0)	0.530
Metabolic disease ≤35 gestational wks	27 (5.3)	326 (2.9)	0.002
Metabolic disease >35 gestational wks	26 (5.1)	343 (3.0)	0.007
Infectious disease ≤35 gestational wks	164 (31.9)	2793 (24.8)	<0.0001
Infectious disease >35 gestational wks	79 (15.4)	1270 (11.3)	0.003
***Clinical-course and medical-care related***			
Reanimation required	339 (66.0)	3888 (34.5)	<0.0001
Mechanical ventilation required	160 (31.1)	209 (1.9)	<0.0001
O_2_ inspiration fraction required > 60 %	95 (18.5)	96 (0.9)	<0.0001
Continuous positive airway pressure required	24 (4.7)	14 (0.01)	<0.0001
Surfactant administration	49 (9.5)	34 (0.3)	<0.0001
Respiratory complication	35 (6.8)	91 (0.8)	<0.0001
Intraventricular bleeding	20 (3.9)	28 (0.2)	<0.0001
Surgical procedure required	14 (2.7)	10 (0.1)	<0.0001
Medical procedure required	293 (57.0)	609 (5.4)	<0.0001

*Mean ± Standard deviation (SD).

When the risk analysis was focused on early-onset sepsis, the independent risk factors comprised; prematurity, PMR >24 h, abnormal amniotic fluid, placental abnormalities, the emergence of respiratory disease, and the requirement of any medical procedure (Table 3).

**Table 3:** Variables that retained statistical significance in risk for neonatal sepsis after logistic regression

**Variable**	**Early-onset**			**Late-onset**		
	**RR**	**(95 % CI)**	**P**	**RR**	**(95 % CI)**	**P**
***Neonate-related factors***						
Low birth weight (≤2500 g)	0.91	0.63-1.32	0.642	1.34	0.74-2.42	0.326
Prematurity (≤37 wks)	2.19	1.41-3.40	<0.0001	1.35	0.57-3.18	0.487
Product of multiple pregnancy	1.13	0.64-1.98	0.669	2.5	1.14-4.80	0.019
Abnormal amniotic fluid	1.67	1.25-2.23	0.007	----	-----	----
Abnormal umbilical cord	1.21	0.89-1.63	0.207	1.15	0.69-1.92	0.584
Perinatal asphyxia	0.78	0.26-2.32	0.656	5.15	1.92-13.83	0.001
***Maternal-related factors***						
PMR ≥24 h^¥^	3.38	1.80-6.32	<0.0001	2.71	0.48-15.30	0.258
Abnormal placenta	1.71	1.16-2.53	0.007	1.17	0.94-3.22	0.078
***Medical-care and hospital-evolution derived factors***						
Mechanical ventilation	1.00	0.90-1.12	0.863	2.71	1.56-4.69	<0.0001
O_2_ inspiration fraction >60 %	3.21	1.95-5.28	<0.0001	2.85	1.57-5.15	0.001
Continuous positive airway pressure	0.87	0.29-2.60	0.804	3.66	1.30-10.27	0.013
Respiratory complication	42.48	25.53-70.67	<0.0001	16.36	3.39-78.91	<0.0001
Surgical procedure required	2.98	0.62-4.37	0.173	28.97	6.99-120.01	<0.0001
Invasive medical procedure required	3.01	2.13-4.26	<0.0001	12.5	6.37-24.56	<0.0001

RR, Relative Rate; 95 % CI, 95 % Confidence interval; PMR^¥^, Premature membrane rupture.

On the other hand, independent risk factors for late-onset sepsis after logistic regression included postmaturity (≥42 wks), perinatal asphyxia, multiple pregnancy, the emergence of any type of postnatal respiratory disease, and requirement of assisted ventilation, O_2_IF >60 %, positive intermittent pressure, or any other type of medical or surgical procedure (Table 3).

### **Mortality-associated factors**

The results of the univariate analysis comparing all collected data between deceased and surviving neonates with sepsis are depicted in Table 4. Multivariate analysis was conducted on all of these. After Cox proportional hazard analysis, only prematurity, low birth weight, perinatal asphyxia, low Apgar score at birth, and the requirement of assisted ventilation or invasive medical or surgical procedures during the hospital stay emerged as independent factors for mortality (Table 5).

**Table 4:** Comparison of all collected data between deceased and surviving septic neonates (univariate analysis)

**Variable**	**Deceased**	**Surviving**	**P**
	**N = 49 (%)**	**N = 465 (%)**	
***Neonate-related***			
Gender (feminine/masculine)	21/28	212/253	0.41
Gestational age (wk)*	31.3 ± 5.2	34.0 ± 4.1	<0.0001
Birth weight (g)*	1486 ± 867	2130 ± 852	<0.0001
Height (cm)*	38.2 ± 6.2	44.0 ± 5.6	<0.0001
Premature (≤37 wks)	38 (77.5)	153 (32.2)	0.08
Postmature (≥42 wks)	1 (2.0)	4 (0.9)	0.39
Product of multiple pregnancy	7 (14.3)	34 (7.3)	0.08
Abnormal umbilical cord	14 (28.6)	117 (25.2)	0.35
With perinatal asphyxia	9 (18.4)	8 (1.7)	<0.0001
With intrauterine hypoxia	3 (6.1)	7 (1.5)	0.06
Apgar score ≤5	42 (85.7)	232 (49.9)	<0.0001
With fetal distress	13 (26.5)	73 (15.7)	0.04
***Maternal-related***			
Age (yrs)*	27.0 ± 5.8	26.3 ± 5.5	0.42
Low educative level	26 (53.1)	258 (55.5)	0.43
Gestations*	2.3 ± 1.3	1.9 ± 1.2	0.06
Abortions*	0.4 ± 0.7	0.2 ± 0.5	0.01
Non-adequate pregnancy control	30 (61.2)	228 (49.0)	0.07
With threat of abortion during this pregnancy	19 (38.8)	120 (25.8)	0.04
Abnormal placenta	15 (30.6)	71 (15.3)	0.008
Abnormal amniotic fluid	20 (40.8)	139 (29.9)	0.08
Dystocic delivery	16 (32.7)	135 (29.0)	0.35
Cesarean section	32 (65.3)	327 (70.3)	0.28
Premature membrane rupture >12 h	10 (20.4)	158 (34.0)	0.03
Premature membrane rupture >48 h	7 (14.3)	130 (28.0)	0.02
Premature membrane rupture >24 h	5 (10.2)	80 (17.2)	0.14
Amnionitis	0	4 (0.9)	0.66
Preeclampsia ≤35 gestational wks	3 (6.1)	25 (5.4)	0.51
Preeclampsia >35 gestational wks	2 (4.1)	24 (5.2)	0.54
Metabolic disease ≤35 gestational wks	4 (8.2)	23 (4.9)	0.25
Metabolic disease >35 gestational wks	1 (2.0)	25 (5.4)	0.26
Infectious disease ≤35 gestational wks	17 (34.7)	147 (31.6)	0.38
Infectious disease >35 gestational wks	7 (14.3)	72 (15.5)	0.51
***Clinical course- and medical care-related***			
Reanimation required	45 (91.8)	294 (63.2)	<0.0001
Mechanical ventilation required	37 (75.5)	123 (26.5)	<0.0001
O_2_ inspiration fraction >60 % required	23 (46.9)	72 (15.5)	<0.0001
Continuous positive airway pressure required	6 (12.2)	18 (3.9)	0.02
Surfactant administration	16 (32.7)	33 (7.1)	<0.0001
Respiratory complication	1 (2.0)	34 (7.3)	0.13
Intraventricular bleeding	9 (18.4)	11 (2.4)	<0.0001
Surgical procedure required	4 (8.2)	19 (2.2)	0.03
Invasive medical procedure required	48 (97.8)	245 (52.5)	<0.0001

*Mean ± Standard deviation (SD).

**Table 5:** Variables that retained statistical significance for mortality in neonates with sepsis after Cox regression model analysis

**Variable**	**HR**	**95 % CI**	**P**
***Neonate-related***			
Prematurity (≤37 wks)	1.08	1.03-1.14	0.02
Low birth weight (≤2500 g)	1.04	1.01-1.08	0.01
Perinatal asphyxia	2.96	1.43-6.15	0.003
Apgar score ≤5	1.40	1.19-1.76	0.004
**Clinical course- and medical care-related**			
Mechanical ventilation required	1.60	1.19-2.40	0.001
Surgical procedure required	2.85	1.49-5.46	0.002
Invasive medical procedure required	2.07	1.63-2.62	<0.0001

HR, Hazard ratio; 95 % CI, 95 % Confidence interval.

## **Discussion**

The incidence of neonatal sepsis varies among the different geographic areas, the highest being registered in Africa and Asia (23-38/1,000 live births) and the lowest, in countries such as the U.S. and Australia (range, 1.5-3.5 /1,000 live births). In South America and the Caribbean, the incidence of neonatal sepsis ranges between 3.5 and 8.9/1,000 live births [12], while in Mexico, the reported rates range between 4 and 15.4/1,000 live births [9]. Although the neonatal mortality rate has been documented in our southeastern Mexico region, the incidence of sepsis and its associated factors remain unknown. The present study was carried out with a population cohort of 11,790 births with a sepsis incidence of 4.3/1,000 live births. A total of 514 neonates were found with sepsis, of which 87 cases were confirmed by means of blood culture; this low sensitivity of the blood culture can be due to the logistical problems involved in culturing very small quantities of blood [16]. Despite that blood culture is considered the gold standard in the diagnosis of sepsis, it is a technique with a low sensitivity, because under optimal conditions, only 5-10 % of suspected cases of sepsis can be confirmed [2,17,18]. In a retrospective study with 21,771 registries (Mclntire, et al.), only 0.3 % of cases of sepsis were confirmed by blood culture [19]. On the other hand, Cohen-Wolkowiew, et al., in a multicenter study performed between 1997 and 2007 in which 119,130 premature newborns from Intensive care units (ICU) were included, documented that 69 % of these had at least one request for blood culture; however, only 0.4 % were confirmed by culture. These authors suggest that the incidence of sepsis can be low in premature newborns because only a small number achieved confirmation [20]. Due to that the majority of neonates with sepsis were premature (68.1 %), it is difficult to determine whether the diagnosis of sepsis based on clinical signs can be confused with prematurity at birth, given that the specific pathophysiology of the sepsis in the premature neonate includes alterations of the immature myocardium and immaturity of the immunological and autonomous nervous systems [21,22].

In our study, the majority of episodes of sepsis (387/514) were documented during the first 3 days of life (early-onset) with a mortality rate of 5.7 %; furthermore, the isolation of infecting microorganism was low 2.3 % (9 isolations) and *Staphylococcus CoNS* were the most commonly isolated microorganisms, while for late-onset sepsis we documented a lower incidence (127/514), but a higher lethality (21.3 %), *Staphylococcus CoNSStaphylococcus aureus* and *Candida ssp.* were the most frequently isolated microorganisms. Currently in the literature, there is no consensus on temporality and type of sepsis; for example, early-onset sepsis can be defined by a period ranging from birth to 48 h or until 6 days of life; this discrepancy affects the interpretation of the rates reported and renders comparison difficult among the different studies. For example, in a study carried out in Kenya in which early-onset sepsis is considered at ≤3 days of life, the authors documented a mortality of 4 % in early-onset and of 10 % in late-onset sepsis; the most commonly isolated microorganisms were *Klebsiella spp*. and *Citrobacter spp.*[23]. In Panama, the authors reported a mortality of early-onset sepsis of 44 % and one of 22 % for late-onset sepsis, with *Klebsiella spp*. and *Staphylococcus aureus* as the most commonly isolated microorganisms; the authors considered early-onset sepsis as ≤5 days of life [24]. In India, where early-onset sepsis is considered up to ≤6 days of life, the authors found a mortality of 49 vs. 86 % in late-onset sepsis; *Klebsiella spp*. and *Pseudomonas spp*. were the most frequently isolated microorganisms [25]. Thus, some authors have proposed classifying sepsis as a type of transmission without taking temporality into consideration; therefore, early-onset sepsis occurs by vertical transmission and is mainly caused by Gram-negative bacteria that are acquired before or after delivery, while late-onset sepsis takes place by horizontal transmission and is principally associated with Gram-positive bacteria acquired after delivery; its source can be nosocomial- or community-acquired [12,26,27]. Recently, Cooper C, et al. reported that early-onset sepsis is defined within 48 h of births, although definitions in some studies include infection up to 72 h after birth, the health care associated infections rarely occur within this period [28]. In the present work, the most frequently isolated microorganism for early- as well as late-onset sepsis was *Staphylococcus CoNS*. Although this bacterium tends to be normal flora of the skin, it has been suggested that it can be pathogenic in newborns submitted to invasive medical procedures (i.e., venous catherization, endotracheal intubation, prematurity, and a prolonged hospitalization period) [29-31].

It also has been described that *Staphylococcus* genus *CoNS* is associated with inflammatory response evasion [32,33]; resistance to antibiotics is measured by plasmids [34] or quorum sensing [35,36]; it also possesses genetic determinants such as operon-Catabolite activator protein (CAP) and the Gamma-glutamyl peptidase (GGP) gene, which confers greater virulence on the bacterium [37,38]. However, despite all of these virulence factors, and although the hospital has certification in the quality attention processing of patients; we are unable to discard the possibility of contamination during venopuncture, or the presence of microorganisms in moist environmental near patient sources such as room temperature bubble oxygen humidifiers. Even though we used standard guidelines for neonatal study recommendation definitions, our findings should be interpreted in the context of the quality of retrospective studies [21,39].

Prior studies have identified risk factors for sepsis such as prematurity, low birth weight, premature membrane rupture, maternal pyrexia, poor intra- and postpartum hygiene, invasive medical procedures, and hospital stay [3,13,40,41]. Likewise, in our study, sepsis-associated factors comprised prematurity, abnormal amniotic liquid, premature membrane rupture, and invasive medical procedures such as mechanical ventilation, O_2_IF ≥60 %, respiratory complication, and surgical procedure, as has been described in previous studies [42,43] (Table 3). All of these factors are present in early-onset sepsis; notwithstanding this, in late-onset sepsis, we documented additional factors such as postmaturity, multiple-pregnancy product, perinatal asphyxia, and all invasive therapeutic procedures (Table 3). It is noteworthy that in this study, we documented that postmaturity is a late-onset sepsis-associated factor (Relative Risk [RR] 7.7; 95 % CI, 1.4-35.7). In this context, Hákansson, et al. reported that post-term infants with >42 weeks of gestational age had a 1.8 times greater risk of developing sepsis and pneumonia [44]; on the other hand; Cheng, et al. documented that in neonates with >40 gestational weeks, the risk of neonatal damage increases mainly due to meconium aspiration (adjusted Odds ratio [OR], 1.27; 95 % CI, 1.17-1.37), which can condition sepsis development [45]. More detailed studies are required that evaluate the specific risk factors for post-term infants.

When we evaluated the clinical factors associated with poor prognosis in 514 neonates with sepsis, we found that prematurity, low birth weight, perinatal asphyxia, and an Apgar score of ≤5, as well as invasive medical procedures, are mortality-associated factors (Table 5). These results are in agreement with previous reports that associate these factors with poor prognosis and death in the neonate with infection [46-49]. Recently, it has been documented that the incidence of pre-term births (at <37 gestational wks) has increased in many countries worldwide and that this represents a public health problem because >70 % of pre-term infants are found within the range of 34–36 gestational weeks (late-onset pre-term). This is relevant because the majority of epidemiological descriptions for neonatal sepsis are directed toward or limited to neonates with very low gestational age (<33 wks) or very low birth weight. Pre-term neonates with late-onset sepsis are traditionally treated by clinicians as full-term neonates; however, there is evidence suggesting that pre-term neonates with late-onset sepsis have a substantial increase in the risk for morbi-mortality [20]. Cheng, et al. have reported that neonates with 37 gestational weeks have a greater frequency of a low Apgar of <7 at 5 min of life (adjusted OR, 1.69; 95 % CI, 1.59-1.79) and a 5-min Apgar of <4 (adjusted OR, 1.87; 95 % CI, 1.63-2.15) with respect to neonates born between 38 and 39 gestational weeks [45]. It has also been described that neonates of <37 weeks have a greater risk of presenting respiratory complications and of consequently requiring mechanical ventilation, which conditions them for greater susceptibility for sepsis [49,50].

We also acknowledge some limitations of this work. First, this study is of retrospective design, and this might inevitably be complicated by incomplete data due to lost records. Second, diagnoses were provided by different caregivers. Unfortunately, ideal prospective data collection by designated study personnel proves extremely difficult in a developing country with limited resources.

## **Conclusions**

In summary, the incidence of neonatal sepsis in southeastern Mexico was 4.3 % and early-onset sepsis was more common in this population. A different risk profile between early- and late-onset neonatal sepsis was found. Poor outcome and mortality were mainly dependent on prematurity, low birth weight, perinatal asphyxia, low Apgar score, a requirement of assisted ventilation, and invasive medical procedures.

## **Methods**

### **Study design and population**

We performed a retrospective cohort design using the collected data of all newborns admitted to the Neonatology Unit of the General Hospital and the High Specialty Medical Unit of the IMSS in Mérida, Mexico, between 2004 and 2007. These medical facilities include tertiary-care medical units at which nearby 1 million persons receive specialized medical care annually. From 2004 to the present, in these medical wards, a prospective and comprehensive data collection was initiated that was focused on clinical and demographical newborn and maternal features, in addition to hospital disease-evolution data, which were recorded based on World Health Organization (WHO) neonatal study recommendation definitions [39]. All data are continuously registered and updated at the patient’s hospital discharge despite the final outcome.

### **Neonatal sepsis definitions**

As a routine procedure in the Neonatology Unit all neonates having perinatal risk factors for sepsis must undergo blood culture screening. Neonatal sepsis was defined according to Pediatric Sepsis Consensus (PSC) criteria [21]. Confirmed sepsis was defined as microbial recovery from blood or any other biologic material culture in addition to the presence of a clinical or biological syndrome of sepsis. For this study confirmed sepsis was assessed from ≈ 1.0 ml peripherical blood with Peds Plus/F culture vials processed by Bactec 9050 System (Becton Dickinson, Inc., USA); since we selected only blood culture, other body fluids such as CSF, pleural and peritoneal fluid were excluded. A clinical and biological syndrome of sepsis was considered despite having negative cultures when the neonate showed positive findings on clinical examination or laboratory tests [e.g., body temperature ≥38 °C o r ≥36 °C, high - sensitivity C reactive protein (CRP) levels ≥ 0.03 mg/dl, high or low leukocyte count according to age (not related with chemotherapy-induced leukopenia), or <10 % immature neutrophils and blood pressure <2 Standard deviations (SD) of the normal value according to age].

### **Collected data**

Registered data were grouped into the following domains:

Neonate-related: Gender; gestational age in week (wk), weight in gram (g), and height in centimeter (cm) at delivery; prematurity; postmaturity; if the neonate was a product of a multiple pregnancy; or if pathological umbilical cord and Apgar score were registered; or if the neonate experienced perinatal asphyxia, intrauterine hypoxia, or fetal distress;

Maternal-related: Mother’s age; educative level (≤6 low, 7–14 medium and ≥ 15 professional); previous pregnancies, deliveries, or cesarean sections; if during the pregnancy the mother received adequate monitoring, or experienced threat of abortion; if the labor ended in dystocic delivery and cesarean section; if placental or amniotic fluid abnormalities were registered; or if during the pregnancy the mother had major infections, metabolic disease, or preeclampsia-eclampsia (all of the three categorized as appearing before or after 35 weeks of gestation), or if the presence of premature membrane rupture was detected;

*Clinical course and medical care-related:* If neonatal reanimation-assisted ventilation, O_2_IF ≥60 %, Continuous positive airway pressure (CPAP), surfactant, or any other invasive medical (such as pleural drainage, umbilical tract, central venous catheterization, or parenteral nutrition) or surgical procedure were required, or if the neonate developed respiratory disease or intraventricular bleeding.

### **Statistical analysis**

In a first step, the risk for sepsis between neonates with and without sepsis was assessed by mean unpaired Student t test, Analysis of variance (ANOVA); Yates corrected Χ_2_ test, or Fisher exact test, according the scale type of the predictor variables. Subsequently, all variables exhibiting a p level ≤0.10 in univariate analysis were entered (Forward Method) in a logistic regression analysis in which Relative Risk (RR) and 95 % Confidence Interval (95 % CI) were estimated. All statistical analyses were performed using the statistical package SPSS for Windows version 17.0).

Furthermore, we assessed the association between all studied variables and death in neonates with sepsis. In this case, all predictor variables having a p value ≤0.10 in univariate analysis were entered into a Cox proportional hazard model in which the association indicator was the Hazard ratio (HR) and its 95 % CI was also obtained. Significance level was set at 0.05 in all analyses performed.

### **Ethical issues**

The project was approved by the High Specialty Medical Unit Research Committee. Because it was a retrospective collateral analysis, informed consent was not required.

## **Abbreviations**

PSC, Pediatric Sepsis Consensus; H, Hour; Wks, Weeks; G, Gram; Yr, Year; Cm, Centimeter; O2IF, O2 Inspiration fraction; CPAP, Continuous positive airway pressure; ICU, Intensive care units; CRP, C-reactive protein; PMR, Premature membrane rupture; CAP, Catabolite activator protein; GGP, Gamma-glutamyl peptidase; WHO, World Health Organization; INEGI, National Institute for Statistics and Geography; IMSS, Mexican Institute of Social Security; SD, Standard deviations; OR, Odds ratio; RR, Relative risk; HR, Hazard ratio; 95 % CI, 95%Confidence interval.

## **Competing interests**

The authors declare that they have no competing interests.

## **Authors’ contributions**

YLH: Has been involved in design the study, data collection, analysis and drafting the manuscript. EPB was involved in data collection. JAN, JDV and URQ were made substantial contributions to the statistical analysis of data and drafting the manuscript. JRV and NDR have been involved in interpretation of the data and revision of the intellectual content. All authors read and approved the final manuscript.

## **Authors’ information**

YAL: Research Associate, PhD. Unidad de Investigación Médica Yucatán (UIMY), Unidad Médica de Alta Especialidad de Mérida (UMAE), Instituto Mexicano del Seguro Social (IMSS), Yucatán, México. JAN: Research Professor, PhD. School of Medicine. Universidad Anáhuac Mayab, Yucatán, México. JRV: Senior Research, PhD. Departamento de Inmunogenética y Alergia, Instituto Nacional de Enfermedades Respiratorias (INER), México. URQ: Fellow Research at UIMY, UMAE-IMSS. Coordinación de Vigilancia Epidemiológica, Unidad de Salud Pública. IMSS. México. NDR: Pediatrician, Neonatologist. Servicio de Neonatología UMAE-IMSS. Yucatán, México. EPB: Neonatology Medical Student and Fellow at UIMY, UMAE-IMSS. Yucatán, México. Cunero Patológico, Hospital General Regional #1-IMSS, Yucatán, México. JVD: Research Associate, MSc. UIMY, UMAE-IMSS. Yucatán, México.

## Pre-publication history

The pre-publication history for this paper can be accessed here:

http://www.biomedcentral.com/1471-2393/12/48/prepub
